# Genomic prediction of fruit texture and training population optimization towards the application of genomic selection in apple

**DOI:** 10.1038/s41438-020-00370-5

**Published:** 2020-09-01

**Authors:** Morgane Roth, Hélène Muranty, Mario Di Guardo, Walter Guerra, Andrea Patocchi, Fabrizio Costa

**Affiliations:** 1 Plant Breeding Research Division, Agroscope, Wädenswil, Zurich, Switzerland; 2 IRHS, INRAE, Agrocampus-Ouest, Université d’Angers, SFR 4207 QuaSaV, Beaucouzé, France; 3 Department of Genomics and Biology of Fruit Crops, Research and Innovation Centre, Fondazione Edmund Mach (FEM), Via E. Mach 1, 38010 San Michele all’Adige, Italy; 4 Department of Agriculture, Food and Environment (Di3A), University of Catania, Catania, Italy; 5 Research Centre Laimburg, Laimburg 6, 39040 Auer, Italy; 6 Center Agriculture Food Environment, University of Trento, Via Mach 1, 38010 San Michele all’Adige, Italy; 7 Present Address: GAFL, INRAE, 84140 Montfavet, France

**Keywords:** Plant genetics, Population genetics, Plant breeding

## Abstract

Texture is a complex trait and a major component of fruit quality in apple. While the major effect of *MdPG1*, a gene controlling firmness, has already been exploited in elite cultivars, the genetic basis of crispness remains poorly understood. To further improve fruit texture, harnessing loci with minor effects via genomic selection is therefore necessary. In this study, we measured acoustic and mechanical features in 537 genotypes to dissect the firmness and crispness components of fruit texture. Predictions of across-year phenotypic values for these components were calculated using a model calibrated with 8,294 SNP markers. The best prediction accuracies following cross-validations within the training set of 259 genotypes were obtained for the acoustic linear distance (0.64). Predictions for biparental families using the entire training set varied from low to high accuracy, depending on the family considered. While adding siblings or half-siblings into the training set did not clearly improve predictions, we performed an optimization of the training set size and composition for each validation set. This allowed us to increase prediction accuracies by 0.17 on average, with a maximal accuracy of 0.81 when predicting firmness in the ‘Gala’ × ‘Pink Lady’ family. Our results therefore identified key genetic parameters to consider when deploying genomic selection for texture in apple. In particular, we advise to rely on a large training population, with high phenotypic variability from which a ‘tailored training population’ can be extracted using *a priori* information on genetic relatedness, in order to predict a specific target population.

## Introduction

Fruits undergo a complex series of genetically programmed events contributing to their attractiveness and suitability for human consumption. Amongst the various physiological and physical ripening processes, texture at maturity is arguably the most important and investigated trait, especially for apple. The apple market is highly structured according to fruit texture, because this feature is important for both consumers’ preference^1^ and storage ability^2^.

Although sensory evaluations with trained panelists can be used to assess variation in texture, these measures are highly dependent on the scorer and limited by sample size^3^. As alternative, texture can be dissected and characterized through texture analyzers in a repeatable way^4,5^. The most recent instruments can measure two groups of sub-traits, mechanical and acoustic, suitable to distinguish between firm (based on mechanical sub-traits) and crisp (based on acoustic sub-traits) types of apples. These texture parameters have been already described and validated in apple^4,5^, and have been used in QTL-mapping studies carried out with biparental populations^6^, Pedigreed Based Analysis and Genome-Wide Association Studies^7^. These studies found a complex genetic basis to fruit texture in apple, identifying a large number of QTLs distributed across the apple genome, with the most relevant regions located on chromosome 3, 10 and 16. This genetic complexity is reflected in the regulation of the cell-wall and middle lamella disassembling, a physiological process orchestrated by a myriad of cell-wall modifying enzymes^8^. Such highly polygenic control can hamper the use of marker assisted selection for improving texture. In the QTL-mapping studies carried out to date, a major QTL located on chromosome 10, close to the polygalacturonase locus *MdPG1*, has been identified^9^. This QTL explains a high proportion (about 40%) of the texture phenotypic variance, but still leaves much unexplained variance that could be used to improve this trait. As reported in the above mentioned study^7^, in modern breeding programs the favorable allele at the locus *MdPG1* has been fixed through successive rounds of *ad hoc* crossing and selection. Given the fixation at *MdPG1*, the phenotypic variance of modern families, obtained by crossing valuable parents for texture performance, might now be under the control of other loci with minor effects. However, QTL-mapping approaches cannot detect such small effect loci, suggesting that other methods are necessary for further improvement of apple texture^10,11^.

To overcome this limitation, an alternative approach for genome-assisted breeding known as genomic selection has been introduced (see seminal work by Meuwissen et al.^12^). In contrast to marker assisted selection, genomic selection relies on the prediction of a genetic value for a genotype, taking into account all genome-wide markers, making it especially relevant for complex traits^13^. For establishing genomic selection, genomic predictions are performed considering two sets of genotypes: a training set (TS) of genotyped and phenotyped individuals to train a prediction model, and the validation set (VS), represented by individuals only genotyped, for which genomic estimated breeding values are predicted^13,14^. In principle, the most favorable scenario for genomic predictions (and subsequent genomic selection) is to predict highly heritable traits in a VS highly related to the TS. While trait heritability can be increased (to a certain extent) by more accurate phenotyping, relatedness between VS and TS can be optimized with different strategies. Dedicated approaches and tools have been proposed to address this issue using optimization parameters^15–17^, and algorithms^18,19^. Specifically, two criteria derived from the mixed model equations used in genomic prediction, the mean of the prediction error variance (PEVmean) and the mean of the expected reliabilities, also called coefficients of determination (CDmean), have been proposed and tested to optimize TS composition using the marker data of both the TS and the VS^15,16^. In theory, it could thus be feasible to acquire phenotypic and genotypic data for a highly diverse TS and then in silico select subsets of individuals to produce an optimal TS for a given VS.

Genomic selection has been largely applied in major crops for primary traits such as yield^14^. However, due to the long generation time of perennial tree species, genomic selection would have a great potential for improving breeding efficiency^20^. The accuracy of genomic predictions, an important factor determining genomic selection efficiency, has been assessed in fruit trees such as apple, peach or citrus^21–23^. In apple, genomic selection was only partially addressed for fruit texture via predictions of classical fruit firmness measurements^24–26^ and of sensory evaluations^24^. Importantly, predictions were typically made within a set of 7–20 full-sib families^21,24,25^, which necessitates a large investment for phenotyping a limited range of families. We propose that the design of a more ‘versatile’ training population, such as a diversity panel, would be more efficient to predict traits of several biparental families, and would thus better qualify for the practical use of genomic selection in apple. To the best of our knowledge, in apple this strategy has only been employed to predict texture using aggregated datasets obtained from historical observations, which has a reduced power due to unaccounted environmental effects, thus limiting the precise identification of the associations between genotype and phenotype^27^.

In this study, we predicted 12 acoustic and mechanical fruit traits describing fruit texture in six full-sib families using a germplasm *collection* as a diverse TS. Furthermore, we were able to improve prediction accuracies by optimizing the TS with respect to the VS of interest. In the light of our results, we discuss the feasibility of genomic selection for improving fruit quality through genomics-assisted breeding programs.

## Results

### Fruit texture phenotypic dissection

**Fig. 1:**
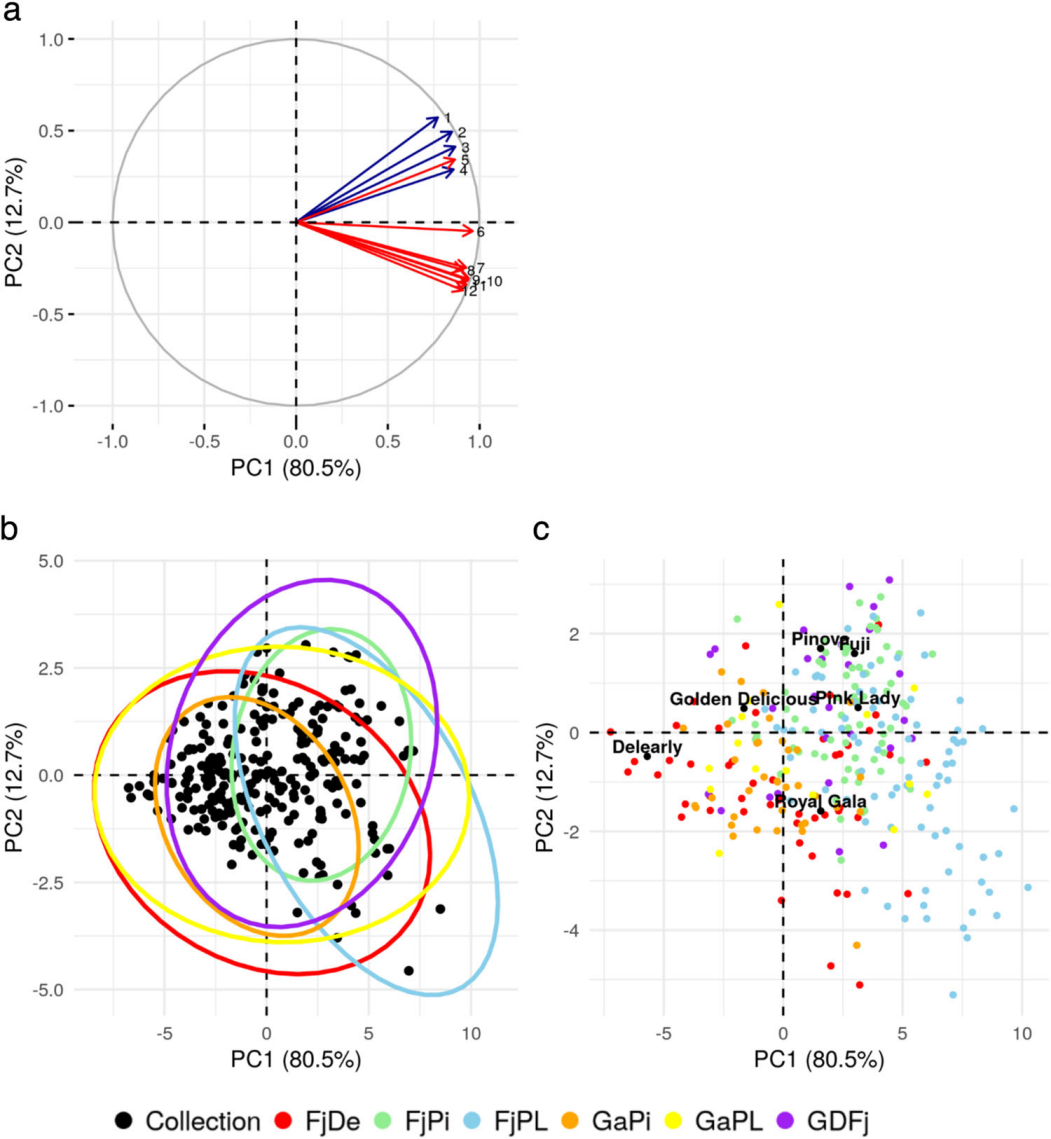
Principal component analysis (PCA) of 12 texture sub-traits using BLUPs of across-year phenotypic values. **a** PCA 2D-plot of variables, with acoustic traits in blue and mechanical traits in red with 1, number of acoustic peaks; 2, acoustic linear distance; 3, acoustic maximum pressure; 4, acoustic mean pressure; 5, number of force peaks; 6, force linear distance; 7, final force; 8, Young module; 9, area; 10; mean force; 11, maximum force; 12, initial force. **b** PCA 2D-plot of individuals with *collection* genotypes represented as dots and families as ellipses. **c** PCA 2D-plot of individuals showing family offspring and their respective parents

The fruit texture phenotypic data used in this survey were represented by four acoustic and eight mechanical sub-traits (Table 1, Table S1). Because of our experimental design, not all environmental effects could be accounted for (tree and genotype effects were confounded, see Methods), and our best linear unbiased predictors (BLUPs) of across-year phenotypic values per genotype approximated the true genotypic values. Assessment of repeatability (defined as the ratio between the variance of across-year phenotypes and total variance) found medium to high values spanning from 0.64 to 0.81 when considering the entire population (*collection* and families) and from 0.59 to 0.75 when considering only the genotypes included in the *collection* (Table 1). With a principal component analysis (PCA) on BLUPs for the 12 textural sub-traits, we identified the main fruit texture profiles, and found contrasted contributions of each trait to variation in fruit texture (Fig. 1a, b). We also found overlapping regions between offspring in biparental families and their parents (Fig. 1c). In this analysis, the PC1 axis, explaining 80.5% of phenotypic variation, and thus comprehensively summarized the general variability of the 12 phenotypic variables. The PC2 axis mainly differentiated the acoustic from the mechanical sub-traits, explaining a smaller, but substantial portion of the phenotypic variability (12.7%, Fig. 1a). However, one mechanical variable, the number of force peaks (FNP), was more correlated with acoustic sub-traits (mean correlation 0.77) than with the rest of the mechanical ones (mean correlation 0.69, Fig. 1a).

**Table 1:** Summary of texture traits assessed in the whole population

Trait	Mean	SD	$\hat R$	$\hat R$_COLL_
ALD	5094	2049	0.751	0.679
ANP	50.4	39.1	0.713	0.630
APMax	65.2	4.38	0.709	0.590
APMean	49.6	3.12	0.795	0.641
Area	813	273	0.798	0.726
FF	10.1	3.98	0.781	0.721
FLD	101	5.78	0.812	0.747
FMax	11.8	4.02	0.783	0.709
FMean	9.6	3.31	0.799	0.723
FNP	17.9	4.16	0.746	0.726
IF	9.94	3.29	0.742	0.659
YM	1.19	0.353	0.637	0.616
PC1	1672.9	564.2	0.782	0.711
PC2	1783.7	714.2	0.730	0.657

For comparison, $\hat R$ values are also given considering measurements in the *collection* ($\hat R$_COLL_). *SD* standard deviation, $\hat R$ repeatability, *ALD* acoustic linear distance, *ANP* number of acoustic peaks, *APMax* acoustic maximum pressure, *APMean* acoustic mean pressure, *FF* final force, *FLD* force linear distance, *FMax* maximal force, *FMean* mean force, *FNP* number of force peaks, *IF* initial force, *YM* young module, *PC1* principal component 1 (synthetic trait), *PC2* principal component 2 (synthetic trait)

Using the texture analyzer, high fruit firmness should be reflected by high mechanical values and high fruit crispness should be reflected by high acoustic values. Based on the interpretation of the distribution of acoustic and mechanical variables in the PCA, different types of texture were identified in the different quadrants (Fig. 1a). Individuals with mealy or soft fruits should be represented by negative PC1 values, while individuals with firm fruits should be represented by positive PC1 values. Besides, individuals with crisp fruits should be more specifically located in the positive PC1 and positive PC2 quadrant, while individuals with firm and non-crisp fruits are expected to fall into the positive PC1 and negative PC2 quadrant (Fig. 1a). Accordingly, as shown by the plot of individuals (Fig. 1b), the distribution of texture profiles on the different quadrant indicated that the *collection* is mainly composed of individuals with low to moderate crispness and firmness (Fig. 1b). It is also important to note that variation on the PC2 axis decreased with decreasing PC1 values, which reflects that high crispness implies a relatively high firmness performance (Fig. 1b).

We found that the six parental genotypes, which are cultivars known to have different texture profiles, were plotted on different quadrants of the PCA 2D-plot (Fig. 1c). ‘Delearly’ and ‘Golden Delicious’ were plotted in the area corresponding to the mealy type of apple, while ‘Royal Gala’ was grouped with moderately firm apples. We found that ‘Fuji’, ‘Pink Lady’ and ‘Pinova’ were positioned in the positive quadrant for both PC1 and PC2, corresponding to the expected crisp type of apple. These positions confirmed the expected texture profile of these six genotypes. The families originating from crossing these genotypes were also distributed over the PCA plot with specific orientations (Fig. 1b, c). In particular, ‘FjPL’ offspring were mostly projected in the ‘firm area’, while ‘GDFj’ offspring were mostly represented in the ‘crisp quadrant’ (Fig. 1b). Moreover, the segregation of the families was very variable with regard to their corresponding parental profiles (Fig. 1c). While ‘GDFj’ was the only family showing a classic type of segregation (intermediate between the parents), the distributions of the other families were more similar to one of the two parents (‘FjDe’ and ‘GaPi’), with a varying number of offspring showing transgressive phenotypes (‘FjDe’, ‘GaPL’, ‘FjPi’ and ‘FjPL’). In particular, while ‘Fuji’ and ‘Pink Lady’ showed a very similar texture profile on PC1 (2.99 and 3.14, respectively), a larger difference was observed on PC2 (1.6 and 0.51, respectively, Fig. 1c, Table S1). Variation in the texture characteristics of ‘FjPL’ offspring was also observed on the PC2 axis, although with a much broader variation than the difference between ‘Fuji’ and ‘Pink Lady’. Accordingly, apples of this family were generally firm to very firm while having a very low to very high crispness (Fig. 1c, Table S1, Fig. S1).

### Additive relationship and genetic clustering in the population

**Fig. 2:**
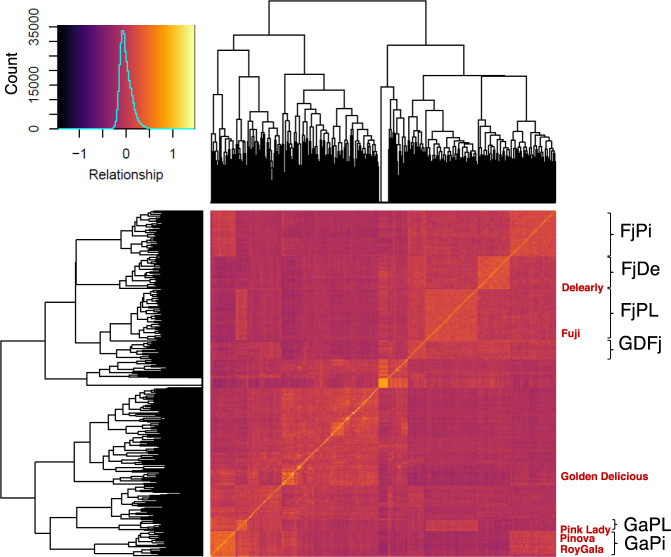
Heatmap representing patterns of relatedness in the population of study. Pairwise realized additive relationship was calculated among the 537 genotypes with 8,294 SNPs, and the distribution of these values appears on the top left corner. The position of families is indicated in black with brackets and the position of parents is indicated with their names in red

In general, the accuracy of genomic prediction is highly correlated to the level of relatedness between the training and the validation sets (TS and VS), and we found here varying levels of relatedness between the *collection* (our TS) and the families (our 6 VS), which can be visualized with a clustering approach and a heatmap on Fig. 2. The parental cultivar ‘Royal Gala’ was found to be the most related to the rest of the *collection* (mean additive realized relationship −6.32 × 10^−4^), while ‘Fuji’ was the most distantly related (mean additive realized relationship −0.102, Table S2). Accordingly, ‘Royal Gala’-related families were more closely related to the *collection* than the four ‘Fuji’-related families (Fig. 2). Mean additive realized relationship values for each family reflected the patterns observed on the heatmap, namely higher values for ‘GaPi’ and ‘GaPL’ (−0.021 to −0.020) and lower for ‘Fuji’-related families (−0.056 to −0.078, Tables 2 and S2).

**Table 2:** Description of the whole population and experimental design used for genomic prediction of texture

						Cluster assignments (# of IDs)						
Name	Mother	Father	Location	Evaluated years	# IDs	1	2	3	4	5	6	Relationship to *collection*
FjDe	Fuji	Delear	FEM	2012–13	50	8	20	0	0	22	0	−0.056
FjPi	Fuji	Pinova	RCL	2012–14	70	1	30	0	0	39	0	−0.078
FjPL	Fuji	Pink Lady	FEM	2012–13	80	0	50	0	0	30	0	−0.071
GaPi	Royal Gala	Pinova	RCL	2012–14	36	0	0	0	0	36	0	−0.021
GaPL	Royal Gala	Pink Lady	RCL	2012–14	15	0	0	0	0	15	0	−0.020
GDFj	Golden Delicious	Fuji	RCL	2012–14	27	0	6	0	0	21	0	−0.057
*Collection*	–	–	FEM	2012–13–15	259	45	37	31	55	66	25	

Maternal and paternal cultivars are indicated for full-sib biparental families. Cluster assignments as given by the discriminant analysis of principal components on 8,294 markers. Relationship to *collection* is the mean additive relationship of progenies relative to *collection*

*FEM* Foundation Edmund Mach, *RCL* Research Center Laimburg

**Fig. 3:**
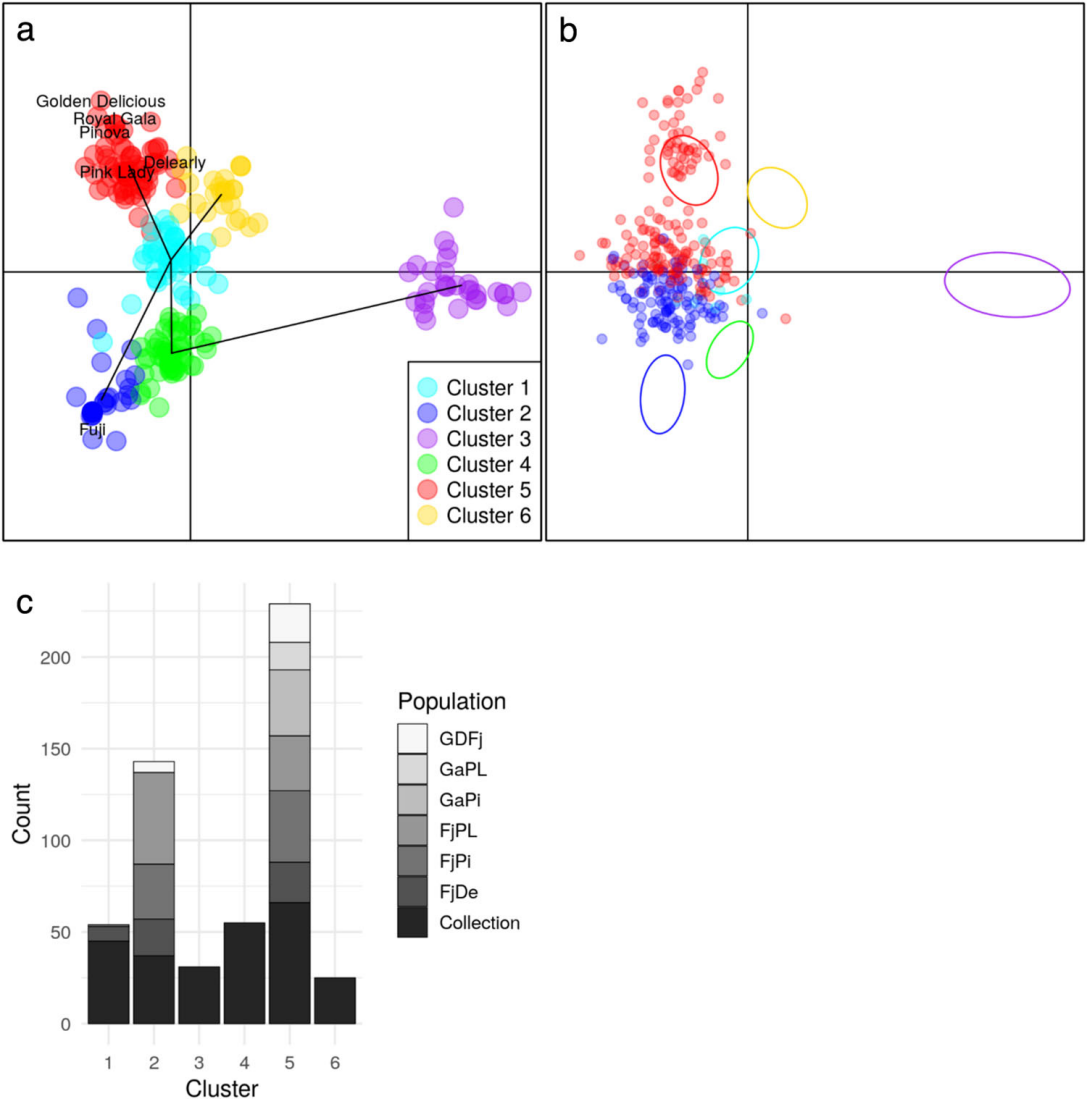
Discriminant analysis of principal components and cluster assignments of genotypes based on 8,294 SNPs. Clusters were identified with a k-means algorithm. **a** Projection on principal component (PC) 1 and 3 of the cluster assignments of genotypes in the *collection* with parents of families indicated with their names. Black lines correspond to the PCs defining clusters. **b** Predicted cluster assignments of progenies of the six full-sib families projected on PC1 and PC3 axes and represented by dots, with *collection* genotypes in the six genetic clusters represented as ellipses (same color legend as in part **a**). **c** Distribution of genotypes across the six genetic clusters in each population

A discriminant analysis of principal component (DAPC) using the entire SNP set (8,294 SNPs) identified the pattern of genetic structure in the *collection*. Using the Bayesian information criterion value (BIC), the most probable genetic structure comprised six clusters and was described by five principal genetic components derived from the marker data (see Methods, Fig. S2). All parental cultivars were assigned to cluster 5, except ‘Fuji’ that was assigned to cluster 2 (Fig. 3a, Table S3). Cluster 5 was the largest group of genotypes (*N* = 66), whilst cluster 6 was the smallest (*N* = 25, Tables 2 and S3). The cluster assignment of the six full-sib families was predicted using the principal components derived by the DAPC analysis carried out on the *collection*. Most of the genotypes were assigned to the parental clusters 2 and 5, although 8 genotypes of ‘FjDe’ and one of ‘FjPi’ were assigned to cluster 1 (Table 2, Fig. 3b, c, Table S3).

Overall, clusters 2 and 5 contained the largest part of the whole population, while clusters 1, 3, 4, and 6 contained the fewest genotypes (Fig. 3c, Table S3). However, while the DAPC analysis suggested this genetic clustering as the most probable in the diversity panel represented by the *collection*, the pairwise Fst-values between clusters indicated low levels of genetic differentiation (values varying between 0.002 and 0.018, Table S4). The Fst value between clusters 2 and 5, containing the parents and most of their offspring, was for instance 0.013. The phenotypic distributions across clusters revealed that individuals assigned to clusters 2 and 5 had elevated values for all traits, except for the synthetic trait PC2, compared to individuals assigned to other clusters (Fig. S3), indicating a possible correlation between genetic clustering and texture traits.

### Cross-validations within the collection

The prediction of marker effects on each trait was obtained with an additive rrBLUP model, where the BLUPs of across-year phenotypic values and the synthetic traits PC1 and PC2 represented the explained variables. The estimated genetic variance, calculated as the variance of the genomic estimated breeding values using this model in the entire *collection*, represented 35–86% of the phenotypic variance, depending on the trait. When running cross-validations within the *collection* with this model on the 14 traits, the highest mean prediction accuracy was obtained for the acoustic linear distance (ALD, *mean cor* = 0.64, Fig. S4), and the second highest accuracy was found for the number of force peaks (FNP, *mean cor* = 0.63, Fig. S4, Table S5). While FNP yielded a relatively high accuracy compared to its repeatability ($\hat R$ = 0.75, Table 1), the overall mean accuracies among traits did not follow the ranking of repeatability obtained within the *collection* phenotypes (Wilcoxon signed-rank-test, *p*-value = 2.44 × 10^−4^). The synthetic traits PC1 and PC2 were moderately predictable with accuracies of 0.59 and 0.42 respectively.

### Genomic prediction in families without training population optimization

In practice, traits can be predicted in families with any available related genetic material that has been genotyped and phenotyped. For this reason, three different scenarios of training population design were tested: either with or without genotypes from the predicted family (‘TS_coll’ and ‘TS_coll_sibs’), or from a half-sib family (‘TS_coll_half-sibs’; see Methods, “Prediction models”), resulting in near-zero to high accuracies, depending on the family, trait, and scenario considered.

**Fig. 4:**
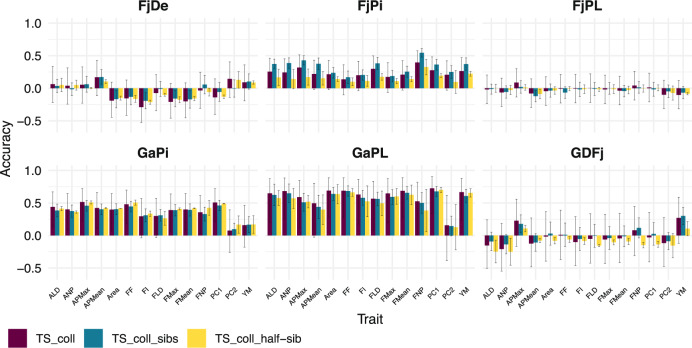
Mean and standard deviation of accuracies obtained with three training population design rules. In ‘TS_coll’ scenario, each family was predicted using only the *collection*. In ‘TS_coll_sibs’ scenario, 30% of genotypes of the predicted family were added to the *collection* in the TS, while the remaining 70% corresponded to the VS. In ‘TS_coll_half-sibs’ scenario, a single half-sib family was added to the *collection* to build the TS

Texture could be predicted with low to high accuracies in three families, ‘FjPi’, ‘GaPi’ and ‘GaPL’; accuracy values ranging from 0.08 for PC2 in ‘GaPi’ to 0.73 for PC1 in ‘GaPL’. Among these three families, the best predicted trait was PC1 (mean for ‘TS_coll’: 0.50, Fig. 4). The three other families showed either accuracies close to zero (‘FjPL’), or negative accuracies (‘FjDe’ and ‘GDFj’, mean accuracies between −0.29 and 0.30, Fig. 4). XY-plots of the observed vs. predicted values for each genotype and for all traits and families are depicted in Fig. S5 with the ‘TS_coll’ scenario.

The addition of related genotypes to the *collection* did not systematically improve predictions. In ‘GaPL’ offspring for instance, predictions were more accurate in the ‘TS_coll’ scenario than with ‘TS_coll_sibs’ and ‘TS_coll_half-sibs’ scenarios, where the TS is enriched with sibs and half-sibs respectively (mean prediction accuracies of 0.60, 0.56, and 0.53 respectively for ‘TS_coll’, ‘TS_coll_sibs’ and ‘TS_coll_half-sibs’ scenarios, respectively). ‘TS_coll_sibs’ particularly improved the accuracies in ‘FjPi’ (mean accuracies of 0.32 in ‘TS_coll_sibs’ vs. 0.26 with ‘TS_coll’), as it better predicted 12 out of 14 traits. ‘TS_coll_half-sibs’ was the lowest performing scenario overall, although it increased the prediction accuracy of seven traits in ‘GaPi’ (increase of 0.01 to 0.09 compared to ‘TS_coll’ scenario, Fig. 4, Table S6).

### Genomic prediction in families with training population optimization

**Fig. 5:**
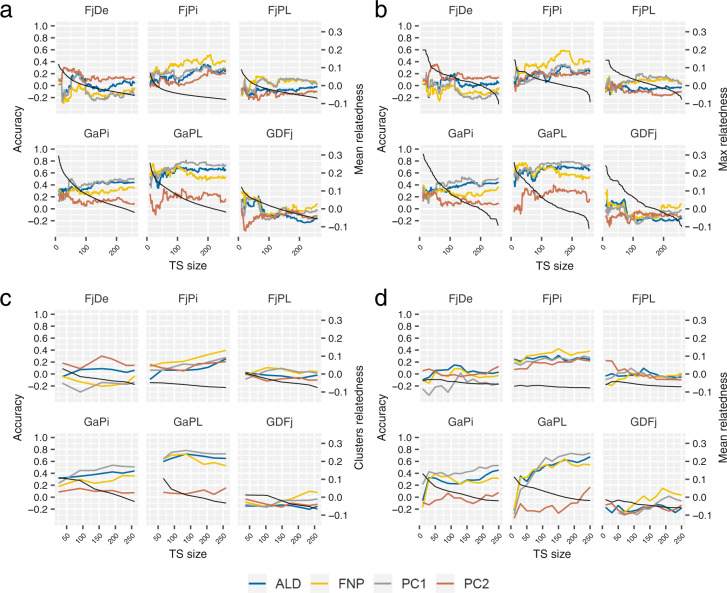
Optimization of the training population for predictions of four texture sub-traits in each family using a priori information from marker data. **a** Addition of genotypes in the TS by decreasing mean relatedness to the predicted family; (**b**) Addition of genotypes in the TS by decreasing maximum relatedness to the predicted family; (**c**) Addition of clusters (identified with a k-means algorithm) by decreasing mean relatedness to the predicted family; (**d**) Selection of genotypes for TS of different sizes based on the five principal components obtained with discriminant analysis of principal components and using the CDmean design criterion. The color legend applies for all parts of the figure. The black curve depicts the value of the relatedness criterion, with the corresponding *y*-axis on the right-hand side. ALD acoustic linear distance, FNP number of force peaks, PC1 principal component 1 (synthetic trait representing firmness), PC2 principal component 2 (synthetic trait representing crispness)

We tested the hypothesis that retaining only the most related genotypes or clusters in the TS might maximize prediction accuracies, and obtained accuracy values for each family and trait using TSs with different sizes and compositions. We observed strong variation in accuracy when genotypes were added in the TS in order of decreasing relatedness until the size of the entire *collection* was reached (Table S7). When considering four traits selected for their practical relevance (ALD, FNP, PC1, and PC2), we also found different accuracy distributions depending on the enrichment criteria used (Table 3, Fig. 5 and Fig. S6). The highest accuracy for each of the 24 (6 × 4) family-trait combinations was in most cases obtained with the addition of single genotypes based on their relationship to the family (in ten cases using the maximum relationship and in ten cases using the mean relationship, Fig. 5a, b, Table 3). The mean optimal population size was 92 genotypes with a minimum size of 10 and a maximum size of 202 genotypes (Tables 3 and S7), meaning that the entire *collection* should not be considered as the optimal TS for predicting texture in the studied families. The maximal accuracies ranged from 0.01 to 0.81, which corresponded to a mean increase in accuracy of 0.17 when comparing to predictions of traits within families using the entire *collection* as TS (minimum increase: 0.02; maximum increase: 0.40 – compared to ‘TS_coll’). The highest accuracy of 0.81 was obtained for PC1 in the ‘GaPL’ family with only 129 genotypes in the TS, i.e., nearly half of the *collection* size. When investigating the distribution of accuracies with increasing TS size in each family for the four focal traits, we observed overall similar trends across traits within a family. In families ‘GaPL’ and ‘GaPi’, which had the highest relatedness to the *collection* (Table 2), the accuracy was moderate to high using as few as 100 genotypes for the traits ALD, FNP and PC1, and remained relatively stable with further increases in TS size (Fig. 5a–d).

**Table 3:** Maximum accuracies obtained among four training set optimization methods in predictions made for each combination of trait and family

Family	Trait	Accuracy	TS size	Method
FjDe	ALD	0.23	77	Mean relationship
	FNP	0.18	21	Max relationship
	PC1	0.26	77	Mean relationship
	PC2	0.36	56	Max relationship
FjPi	ALD	0.36	189	Mean relationship
	FNP	0.59	174	Max relationship
	PC1	0.36	178	Max relationship
	PC2	0.26	202	Mean relationship
FjPL	ALD	0.10	130	CDmean-opt
	FNP	0.16	22	Max relationship
	PC1	0.20	120	Max relationship
	PC2	0.22	10	CDmean-opt
GaPi	ALD	0.46	156	Mean relationship
	FNP	0.40	13	Max relationship
	PC1	0.54	191	Clusters
	PC2	0.28	19	Mean relationship
GaPL	ALD	0.72	136	Clusters
	FNP	0.78	37	Max relationship
	PC1	0.81	129	Mean relationship
	PC2	0.40	140	Max relationship
GDFj	ALD	0.21	66	Mean relationship
	FNP	0.32	15	Max relationship
	PC1	0.19	31	Mean relationship
	PC2	0.01	10	Mean relationship

*TS* training set, *ALD* acoustic linear distance, *ANP* number of acoustic peaks, *FNP* number of force peaks, *PC1* principal component 1 (synthetic trait), *PC2* principal component 2 (synthetic trait)

‘FjPi’ was the only family for which increasing TS up to 200 genotypes resulted in a clear accuracy improvement, and this result was consistent across the four approaches we implemented (Fig. 5a–d). In families with low overall accuracies, such as ‘FjDe’, ‘FjPL’ and ‘GDFj’, the highest accuracy was in most cases obtained with as few as 10–70 genotypes and declined or remained stable with increasing TS size (Fig. 5a–d). In ‘GDFj’, for example, accuracies above 0.2 were found with a TS of 10–66 genotypes (Fig. 5a–c, Table S7). Moreover, while FNP was not predictable in ‘GDFj’ with the entire *collection* (*cor* = 0.08 for *N* = 259), an accuracy of 0.32 was observed with as few as 15 highly related genotypes (based on maximum relationship, Fig. 5b).

## Discussion

### Family-dependent fruit texture profiles and fruit texture prediction

The twelve texture “sub-traits” showed moderate to high repeatability and the magnitude of genetic variation in traits differed between families, with frequent transgressive segregation patterns. The lower repeatability obtained for traits in the collection could be explained by the larger biological variation caused by the sampling of five fruits across trees (three trees/genotype, i.e., 1.67 fruit/tree), in contrast to genotypes of the families for which the five fruits were sampled within single trees (and where the tree effect is confounded with the genotypic effect). Using BLUPs and marker data only, we could predict texture features with moderate to high accuracy within the *collection* (accuracies between 0.42 and 0.64), which indicates that, despite the experimental and statistical limitations imposed by our design (pseudo-replication, different sampling design in families and *collection* orchards), substantial genotypic effects were assessed with our approach.

The *collection* was further used as main training population to predict texture in families. As we have observed, prediction accuracies were quite heterogeneous between biparental families. Without TS optimization, texture could be accurately predicted for ‘GaPL’ (mean accuracy of 0.57), while ‘GaPi’ and in ‘FjPi’ showed a moderate prediction accuracy (mean accuracy of 0.30). In contrast, near-zero or negative accuracies were obtained for ‘FjDe’, ‘FjPL’ and ‘GDFj’ across all traits (mean accuracy of −0.05). The large negative accuracy values repeatedly obtained in ‘FjDe’ and ‘GDFj’ could reveal an opposite linkage phase at markers closely linked to the relevant QTLs between these families and the *collection*^28^. They could also reflect a systematic bias caused in the calculation of the Pearson correlation coefficient itself, in particular when the means of the TS and the means of the VS for the predicted trait are negatively correlated over the sampling procedure^29^. The low accuracies suggest that genomic selection for fruit texture in families ‘FjDe’, ‘FjPL’ and ‘GDFj’ would be ineffective using the present experimental design.

Our results also highlight considerable variability in the prediction accuracies across texture components. In particular, we found large differences in accuracy between firmness (as approximated by PC1) and crispness (as approximated by PC2), the two main components of texture dissected with our PCA (Fig. 1). PC1 was among the most predictable traits (accuracy of 0.59 in *collection* and highest accuracy among traits and family: 0.73 in GaPL), while PC2 showed generally low prediction accuracies. The high phenotypic variability explained by PC1 in the collection (80.5% of total phenotypic variability, while PC2 accounted only for 12.7%, Fig. 1), together with a higher repeatability (Table 1), are both factors likely to contribute to these higher accuracies in PC1 relative to PC2. As a comparison, our repeatability estimates are in line with the medium to high heritability obtained when measuring firmness with texture analyzers and the low to medium heritability obtained in sensory evaluations for crispness^24,25,30,31^.

The prediction design and strategy we adopted involves the phenotyping of one single training population (*collection*) to predict texture within multiple families. Previous works on texture prediction have mainly focused on firmness, and generally showed low accuracies when predicting unobserved genotypes in a set of families or in a *collection* (between 0.15 and 0.35)^24,26,27^. A much higher accuracy of 0.83 was found for firmness in the work of Kumar et al.^25^ when performing cross-validations within a 4 × 2 factorial design, with 1080 and 120 genotypes randomly assigned to the TS and VS, respectively. This strategy is expected to yield higher prediction accuracies, but phenotyping such large numbers of genotypes to make accurate predictions for only a few families would be unfeasible in a commercial breeding program. Moreover, relying on a training population derived from a small number of parents makes such an approach less suitable for making predictions across a broad range of breeding material. To this end, the design we proposed is more versatile and enables the user to share the costs of phenotyping the TS on a larger scale, which should be more suitable for the practical use of genomic selection.

Beyond firmness, we also investigated variables highly correlated to sensory crispness, a trait which strongly influences the sensory experience of consumers and thus determines the commercial success of a cultivar. To our knowledge, predictions for crispness have only been reported for sensory crispness with an accuracy around 0.2^24^. In the populations considered in our study, crispness (as obtained by PC2) could be predicted with a reasonable accuracy of 0.42 in the *collection*, and in most of the families we could achieve accuracy values above 0.2 (except family ‘GDFj’, with 0.01 maximum accuracy).

### Impact of genetic clustering and relatedness on prediction accuracy

Through the implementation of the DAPC method, six significant, yet marginally differentiated genetic clusters were observed. Families were assigned to one or two specific clusters, reflecting the assignment of their parental genotypes. Our results confirm the weak genetic structure characteristic of the cultivated apple^32,33^. Although some degree of correlation was apparent between the genetic clustering of genotypes and their phenotypic distribution (Fig. S3), TS optimization based on clustering was the lowest performing among the four methods that we tested. One important result revealed by the clustering patterns was that the two families ‘GaPL’ and ‘GaPi’, whose parents originated from the same highly represented genetic cluster in the *collection* (Cluster 5), yielded the best predictions.

The genetic parameter having the largest impact on predictions was genetic relatedness, with texture traits being much better predicted in the two families most related to the *collection* (‘GaPL’ and ‘GaPi’) compared to the remaining ‘Fuji’-related families. This observation confirms that genetic relatedness is a fundamental parameter in genomic prediction^34^. The addition of closely related genotypes from the same family (‘TS_coll_sibs’) or from a complete half-sib family (‘TS_coll_half-sibs’) to the *collection* did not improve the prediction accuracy for five of the six families studied. This result suggests that either the *collection* already contains ‘enough’ diversity to predict families, or that the excess of unrelated genotypes in the *collection* cannot be corrected by adding more related genotypes. Thus, ‘TS_coll_sibs’ and ‘TS_coll_half-sibs’ scenarios do not seem to effectively improve the TS.

To resolve this uncertainty, we used an alternative optimization strategy, which involved gradually increasing the TS size using *a priori* information on genetic relatedness derived from marker data. TS optimization based on *a priori* information on relatedness between genotypes improved the accuracy of predictions relative to other composition rules ‘TS_coll’, ‘TS_sibs’, ‘TS_half-sibs’, with a minimal increase of 0.2 and maximal increase of 0.4 in accuracy (Figs. 5 and S6, Tables 3 and S7). Importantly, the maximum accuracies were never reached by using the entire set of 259 genotypes as TS, especially for families with the lowest genetic relatedness to the *collection*. For genomic selection, it is usually recommended to use a large and diverse training population^35^ that includes at least one closely related genotype in the TS for each genotype in the VS^36^. However, in the present study this was not sufficient to maximize accuracies when genotype with low relatedness to the VS were retained for training the model. Our results are consistent with previous findings in barley^37^ showing the detrimental effects of adding genotype unrelated to the VS into the TS. In the present design, highly related ‘*ad hoc*’ training populations are more suitable for predicting biparental populations than larger ones where mean relatedness is reduced, a finding that has also been reported in maize biparental populations^38^.

### Improving the genomic selection strategy for apple texture

The improvement of fruit texture is still limited by the time-consuming and expensive process of phenotyping with texture analyzers. Thus, even though our predictions demonstrate the potential of genomic selection for apple texture, its practical application will be profitable if accurate predictions can compensate for the costly and laborious phenotyping of the TS. Texture analyzers should be preferred over sensory assessments because their measurements are highly repeatable, giving higher heritability estimates for texture traits^5^. To make its use more affordable and appropriate for real breeding programs dealing with high number of genotypes, one possibility could be to select a single but fundamental parameter to measure. In this regard, the mechanical trait FNP, the number of mechanical peaks observed in the mechanical profile generated by fruit compression on the texture analyzer, was found to be highly correlated to acoustic variables and associated to crispness (PC2) in this study. As mechanical traits are easier to measure than acoustic ones, FNP could in practice replace acoustic traits to assess crispness. Since we also obtained high prediction accuracy for FNP (0.63 in *collection* and maximum of 0.78 in optimized family prediction), we propose this sub-trait as the most valuable descriptor for fruit texture, minimizing the effort needed to phenotype that trait.

Identifying the principal components of texture profiles allowed us to capture the fruit texture phenotypic variability hidden within the twelve measured sub-traits. We exploited the high correlations between sub-traits and PC1 to facilitate and improve the prediction of firmness. This simplified multiple-trait approach could be further exploited by using proper multiple-trait models (see for instance in rye^39^, where the prediction accuracy of protein content was improved by using a two-trait model involving yield).

In the experimental design presented here, the performance of genomic predictions and thus the applicability of genomic selection for texture depended highly on the target family, and more generally on the relatedness between TS and VS. Considering the constrained resource allocation proper to the design of fruit trees experiments, we propose three strategies ranked by order of priority for increasing prediction accuracies towards the application of genomic selection, which are (i) to increase marker density to better harness relatedness at causal loci and address potential linkage phase inversions between TS and VS; (ii) to broaden the genotypic diversity of the main TS to better represent the material of interest for breeding (especially for crispness); (iii) to increase the heritability estimates for fruit texture by assessing this trait in the TS using both more replicates and different environments. In the future, the use of reference populations (or so called “REFPOPs”) with high replication, high density genotyping, and high genetic diversity should help address these limitations^40,41^.

## Materials and methods

### Plant material

The plant material and phenotyping strategies used in this work have been described previously^5–7^. Two types of plant materials were used in this study: a *collection* of apple genotypes, expected to represent the diversity in the cultivated apple and thus serving as main TS, and six biparental families, which are typical examples of the material used for selection in apple breeding programs, thus serving as six different VS. The apple *collection* consisted of 259 distinct genotypes, each represented by a single plot of three adjacent trees (clones), at the experimental orchard of the Fondazione Edmund Mach (Trento) in the Northern part of Italy. The six biparental families contained a total of 278 genotypes. Two (‘FjDe’: ‘Fuji’ × ‘Delearly’ and ‘FjPL’: ‘Fuji’ × ‘Pink Lady’) were located at the Fondazione Edmund Mach (in the same orchard as the *collection*), while the other four (‘GaPL’: ‘Royal Gala’ × ‘Pink Lady’, ‘GaPi’: ‘Royal Gala’ × ‘Pinova’, ‘FjPi’: ‘Fuji’ × ‘Pinova’ and ‘GDFj’: ‘Golden Delicious’ × ‘Fuji’) were planted at the experimental orchard of the Laimburg Research Center (Bolzano), located in the same area with near-identical climatic and pedological conditions. In contrast to the *collection*, each genotype from the six families was represented by a single tree. At the time of the analysis, all plants (from both the *collection* and families) were in a productive and adult phase. Fruit texture was phenotyped in 2012, 2013, and 2015 for the *collection*, in 2012 and 2013 for ‘FjDe’ and ‘FjPL’, and in 2012 and 2014 for the four remaining families (Table 2). Plants from both the *collection* and families, were grafted on ‘M9’ rootstock and grown according to conventional horticultural management for plant training system, pruning, and pest-disease control.

Fruits were harvested from each genotype at the physiological ripening stage, established according to standard horticultural fruit quality parameters, such as the change in color of the skin, seeds and flesh, fruit firmness value and the iodine coloration index indicating the level of internal starch degradation. Fruit from different trees of the same plot were harvested the same day. After harvest, fruits were stored for 2 months at 2 °C with 95% relative humidity.

### Texture phenotyping

The texture of the apple fruit was assessed via mechanical and acoustic measurements with the use of a texture analyzer TA.XT*plus* (Stable MicroSystems Ltd., Godalming, UK) equipped with an acoustic envelop device AED (Stable MicroSystems Ltd., Godalming, UK), as previously described^5^. From the harvested apples, a single, homogeneous set of five apples was chosen for each genotype (i.e., the set of five apples came from a single tree for families and from different trees for the collection). Four identical discs were taken per fruit, avoiding seeds, seed cavity tissues or skin, for a total of 20 measurements per genotype (five biological replicates, and four technical replicates per biological replicate). Each texture profile was then digitally converted to 12 texture measurements (i.e., ‘sub-traits’), four related to the acoustic performance and eight to the mechanical force-displacement. In brief, the mechanical sub-traits were coded as: initial, final, maximum, and mean force (related to the different force values associated with different parts of the force-displacement profile), area, force linear distance (derived length of the profile), Young’s module (also known as elasticity module) and number of force peaks. The four acoustic sub-traits were the maximum measure of acoustic pressure, the mean of acoustic pressure measures, the acoustic linear distance and the number of acoustic peaks obtained with the texture analyzer. A more detailed description of the texture sub-traits has been previously reported^5^.

### SNP genotyping

The DNA used for SNP-genotyping in this survey was extracted from young leaves collected from a tree of each genotype at the beginning of the vegetative phase with the Qiagen DNeasy Plant Kit and further quantified with a Nanodrop ND-8000 (ThermoScientific, USA). The SNP marker data were obtained with the HiScan (Illumina, USA) and the apple 20K SNP chip Infinium array (Illumina, USA); the chip was assembled within the framework of the European project FruitBreedomics^42^. The genotyping output was initially analyzed with the software GenomeStudio and further re-edited with ASSiST^43^. SNPs with minor allele frequencies lower than 0.05 and call rate below 0.2 were filtered out with the package ‘snpStats’^44^. The final set of markers successfully recovered in the population consisted of 8,294 biallelic SNPs.

### Analysis of the fruit texture sub-traits

For each trait, we used a mixed linear model to get the BLUP of across-years phenotypic value for each genotype. These BLUPs were used to explore the phenotypic distributions via a PCA and were subsequently employed in the two-step genomic prediction scheme described below (‘Prediction models’ section). We first calculated the mean of the four technical replicates for each apple to retain only the biological replication level in the model (i.e., a single apple). For genotypes of the collection, the biological variation represented by individual apples encompasses the variation within and between trees of the same genotype. However, it was not possible to account for the effect of individual trees as apples were bulked without recording the specific tree identifiers. Variation in each of the 12 mechanical or acoustic sub-traits, considered as ‘Y’, was modeled using the genotype as a random effect, the trial (location-by-year) as fixed effect and a random effect of error: $Y_{i,j,k} \,=\, \mu + {\mathrm{genotype}}_i + {\mathrm{trial}}_j + e_{i,j,k}$ (1). Each phenotypic datapoint *Y*_*i,j,k*_ is explained by the mean *μ*, the genotype i, the trial j and the error for each combination of genotype, trial and replicate (k, i.e., a single apple). To note, apples correspond here to pseudo-replications of the genotype levels and the across-year phenotypic values represented by BLUPs encompass the effect of individual trees. This model was fitted separately for all traits with the ‘lme4’ R-package^45^. As our design did not allow to calculate heritability precisely, the repeatability was calculated instead, as defined by the ratio between the variance of genotypes as assessed by across-year phenotypes and the total phenotypic variance.

A PCA was performed on BLUPs with the ‘FactorMiner’ R-package^46^. The values from the *collection* were used to create the principal components, while the individuals from the families were plotted as supplementary individuals. Coordinates of individuals on the first and the second PCs (‘PC1’ and ‘PC2’) were used for prediction and named ‘synthetic’ traits. The loadings of PC1 and PC2 were used on the raw, replicated data to compute the repeatability estimates of both synthetic traits.

### Kinship and clustering analyses

The realized additive relationship^47^ was calculated with the entire SNP dataset with the VanRaden method described in ref. ^48^ implemented in the ‘A.mat’ function of the ‘rrBLUP’ package^49^, and depicted in a heatmap plot using the R-function ‘heatmap.2’ (package ‘gplots’^50^). Genetic clustering was further assessed in the *collection* to identify potential genetic groups having an impact on the prediction results for texture. To this aim, we performed a DAPC^51^, carried out with the R-package ‘adegenet’^52^ using the entire set of 8294 markers. In the first step, six significant clusters were retained with the function ‘find.clusters’ using 300 principal components and selecting the number of clusters with the highest likelihood (based on the BIC, Fig. S1). Out of these principal components, 150 were retained and employed in the clustering computed with the ‘dapc’ function, which created five principal components that maximized the distance between clusters while minimizing the distance between genotypes within each cluster. The assignment of offspring to clusters was obtained with the function ‘predict_dapc’. Pairwise *F*_ST_ values between clusters were then computed with the entire SNP set using the function ‘pairwise.WCfst’ from the R-package ‘hierfstat’^53^.

### Prediction models

Genomic predictions were computed through a model implemented in the rrBLUP framework^49^, $Y \,=\, \mu + Zu + e$ (3). Y is the vector of BLUPs of the across-year phenotypic values (*n* × 1), *μ* is the mean of the phenotype, Z is the *n* × *p* incidence matrix linking the marker data (additive coding −1,0,1) to observations of Y, *u* the p × 1 vector of random marker effects with $u \sim N\left( {0,\,I\sigma _u^2} \right)$, and *e* is a *n* × 1 vector of random errors. For the predictions of marker effects, the incidence matrix Z contained the observations from the TS, and observations from the VS were masked. In a second step, the predicted marker effects were employed to obtain the genomic estimated breeding values in the VS.

While the ultimate goal was to predict texture in biparental families with the *collection* as main TS, we first performed fivefold cross-validations, repeated 100 times, within the *collection*. These results served as a baseline to interpret the subsequent predictions of texture in families, using the *collection* (or part of it) as TS.

When predicting texture within each family (considered as VS), different TS composition rules were tested. In the first approach, the design was made using the information on relatedness known before genotyping between TS and VS (i.e., without using marker data). There, we used three composition rules: in scenario ‘TS_coll’, the simplest case, each family was predicted using the *collection* only. In scenario ‘TS_coll_sibs’, 30% of genotypes of the predicted family were added to the *collection* in the TS, while the remaining 70% formed the VS. In scenario ‘TS_coll_half-sibs’, a single half-sib family (e.g., ‘GaPL’ is half-sib with ‘FjPL’ and ‘GaPi’) was added to the *collection* to form the TS, leading to two to four TS possibilities (and accuracy values). To give an example focusing on the family ‘GaPi’, we tested the three following scenarios: scenario ‘TS_coll’ corresponded to [TS = *collection* // VS = ‘GaPi’] (one accuracy estimation only); ‘TS_coll_sibs’ corresponded to [TS = 30% ‘GaPi’ offspring + *collection* // VS = 70% remaining offspring of ‘GaPi’] (sampling of the 30% repeated 100 times, giving 100 estimations of the accuracy); and ‘TS_coll_half-sibs’corresponded to [TS = ‘GaPL’ *or* ‘FjPi’ + *collection* // VS = ‘GaPi’] (resulting in the estimation of two accuracy values).

In the second approach, we performed TS optimization with the use of information on genetic relatedness between TS and VS as inferred by marker data. There, we looked for the optimal TS size and composition with a relatedness-driven and a principal component-driven approach. The relatedness-driven approach was tested in three different ways: (i) by starting with the ten most-related genotypes and adding single genotypes with decreasing mean relationship to the family; (ii) with decreasing maximum relationship to the family (*N* = 10 to *N* = 259 for (i) and (ii)); or (iii) by starting with a TS composed of the most related cluster and adding less and less related clusters successively (final TS size *N* = 259). In the principal component-driven approach, TS individuals were selected to constitute several TS with increasing size using the ‘CDmean’ criterion to choose individuals (R-package ‘STPGA’^54^). The CDmean criterion utilizes the generalized coefficient of determination between contrasts of genotypes^16^, as a measure of the reliability of prediction, to build the optimal TS via an iterative process. This criterion was selected because it should allow maximizing prediction accuracy without decreasing the genetic variance in the TS^15,17^. Specifically, the optimal TS was allowed to vary from 10 genotypes to 259 in increments of 20 genotypes, and was chosen based on the five principal components obtained with DAPC analysis with the algorithm implemented in the function ‘GenAlgForSubsetSelection’ (R-package ‘STPGA’^54^). Here, genotypes were chosen independently for each TS size, meaning that we did not proceed to a gradual enrichment of the TS.

All accuracy values were based on Pearson correlation coefficients calculated between observed values (i.e., BLUPs of across-year phenotypic values) and predicted values of the VS genotypes. When standard deviations were not available, we calculated an ~95% confidence interval of the correlation coefficient with a Fisher’s Z-transformation (‘cor.test’ function in base R). Calculations were performed in R statistical software^55^ and graphs were created with the R-package ‘ggplot2’^56^. The R-scripts used for this study are available at https://github.com/MorganeRoth/GS_apple_texture.

## Supplementary information

Supplementary Figures 1 to 6

Table S1. Texture genotypic values and coordinates for PC1 and PC2

Table S2. Additive relationship matrix

Table S3. Assignments of individuals to genetic clusters

Table S4. Pairwise Fst-values between genetic clusters

Table S5. Accuracies obtained in cross-validations within the collection

Table S6. Accuracies obtained in family predictions using two models and three TS scenarios

Table S7. Accuracies obtained in family predictions with TS optimization with four methods
