# Supplementary material for: Genomic prediction of fruit texture and training population optimization towards the application of genomic selection in apple
Source: Hortic Res. 2020 Sep 1;7:148. doi: 10.1038/s41438-020-00370-5 (PMC7459338; doi:10.1038/s41438-020-00370-5)
Supplement: Supplementary file 5 — Table S4. Pairwise Fst-values between genetic clusters [file 41438_2020_370_MOESM5_ESM.pdf]

Table S4. *Pairwise Fst values between clusters identified via a discriminant principal component analysis. Values calculated in the entire population with 8,294 SNP markers*

**Clusters**	**1**	**2**	**3**	**4**	**5**	**6**
**1**	NA	0.012	0.002	0.003	0.002	0.003
**2**	0.012	NA	0.014	0.018	0.013	0.018
**3**	0.002	0.014	NA	0.003	0.002	0.008
**4**	0.003	0.018	0.003	NA	0.007	0.005
**5**	0.002	0.013	0.002	0.007	NA	0.007
**6**	0.003	0.018	0.008	0.005	0.007	NA
