# Supplementary material for: Genomic prediction of fruit texture and training population optimization towards the application of genomic selection in apple
Source: Hortic Res. 2020 Sep 1;7:148. doi: 10.1038/s41438-020-00370-5 (PMC7459338; doi:10.1038/s41438-020-00370-5)
Supplement: Supplementary file 6 — Table S5. Accuracies obtained in cross-validations within the collection [file 41438_2020_370_MOESM6_ESM.pptx]

## Slide 1
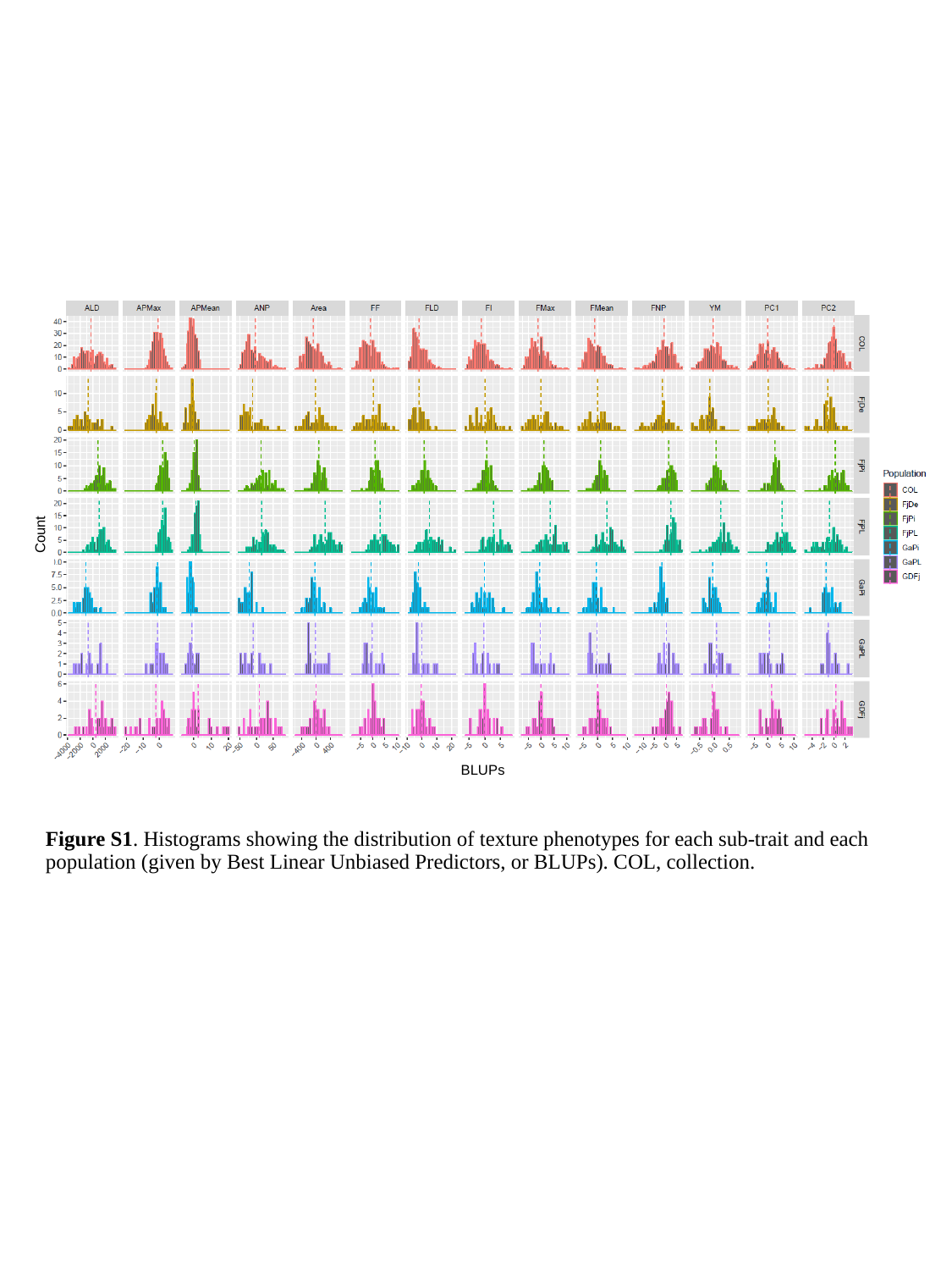

Count
BLUPs
Figure S1. Histograms showing the distribution of texture phenotypes for each sub-trait and each population (given by Best Linear Unbiased Predictors, or BLUPs). COL, collection.

## Slide 2
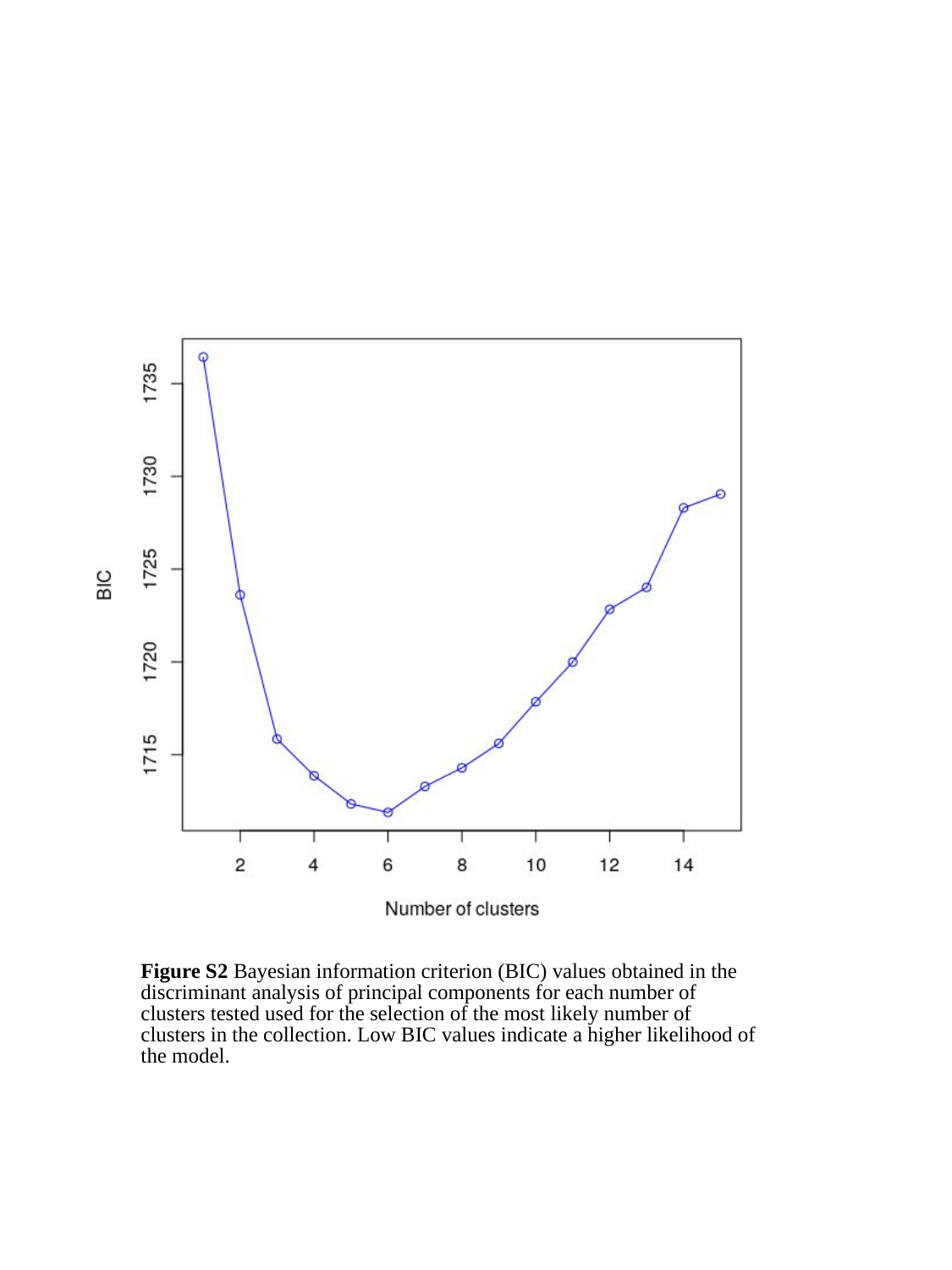

Figure S2 Bayesian information criterion (BIC) values obtained in the discriminant analysis of principal components for each number of clusters tested used for the selection of the most likely number of clusters in the collection. Low BIC values indicate a higher likelihood of the model.

## Slide 3
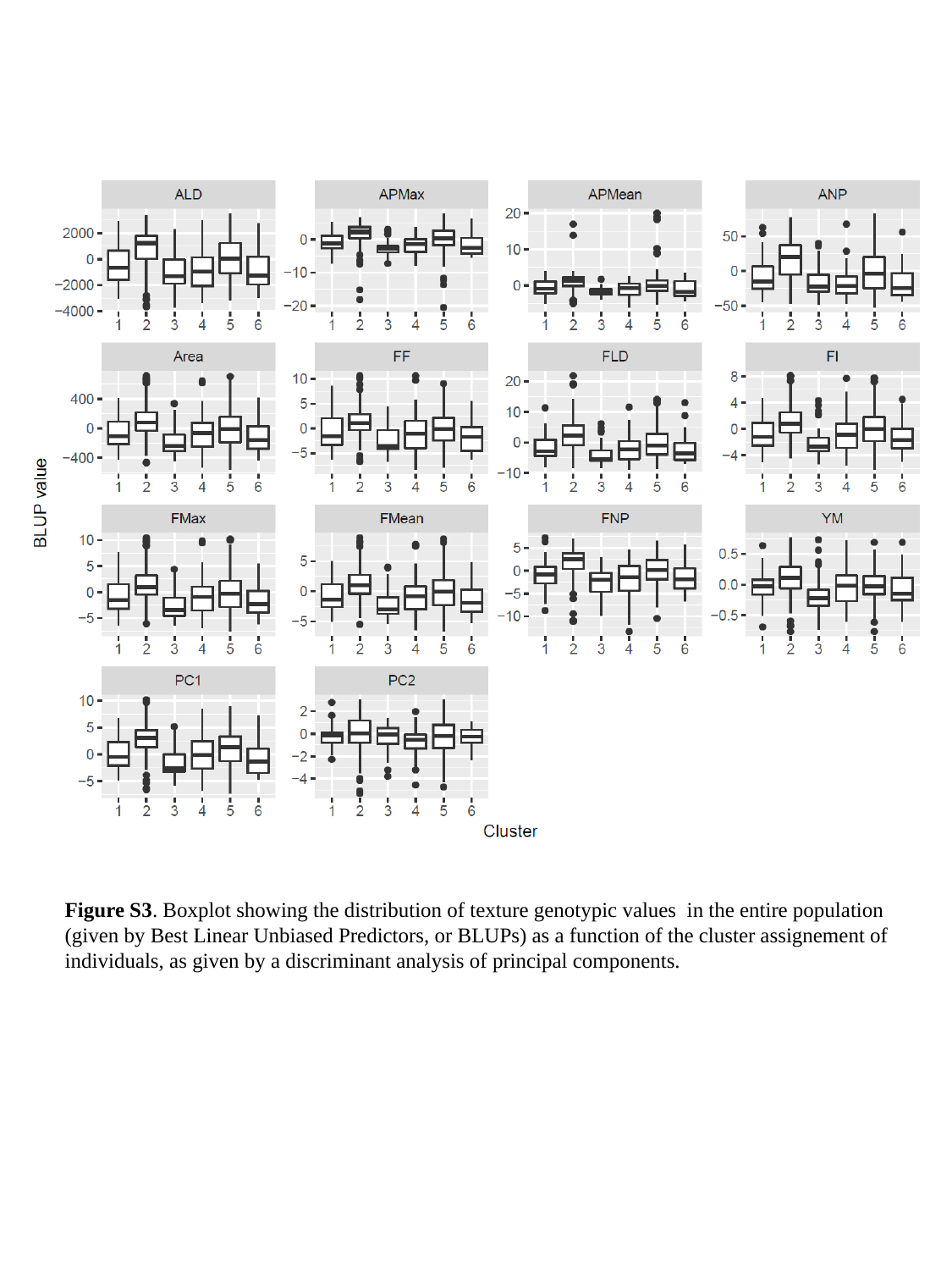

Figure S3. Boxplot showing the distribution of texture genotypic values in the entire population (given by Best Linear Unbiased Predictors, or BLUPs) as a function of the cluster assignement of individuals, as given by a discriminant analysis of principal components.

## Slide 4
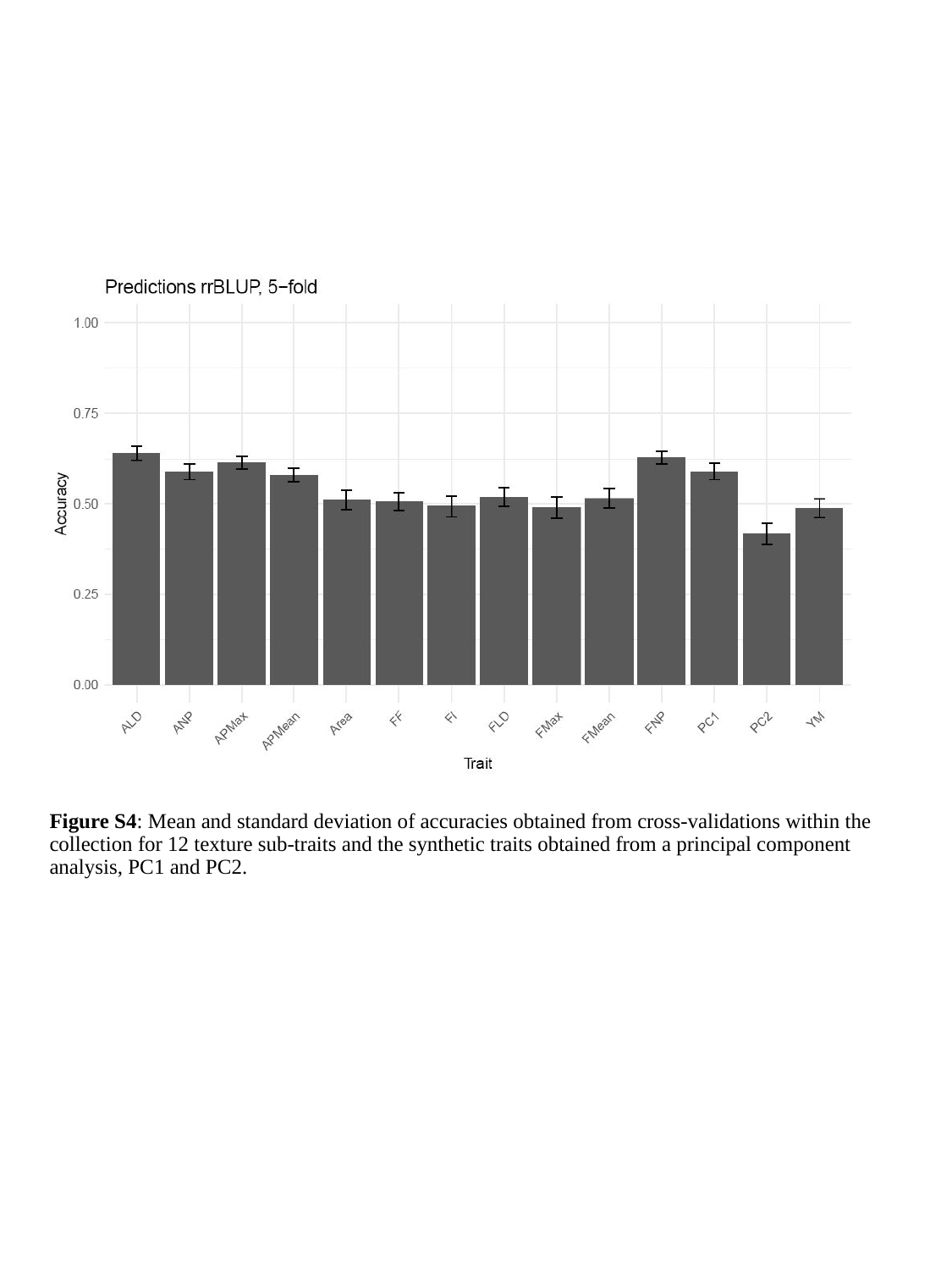

Figure S4: Mean and standard deviation of accuracies obtained from cross-validations within the collection for 12 texture sub-traits and the synthetic traits obtained from a principal component analysis, PC1 and PC2.

## Slide 5
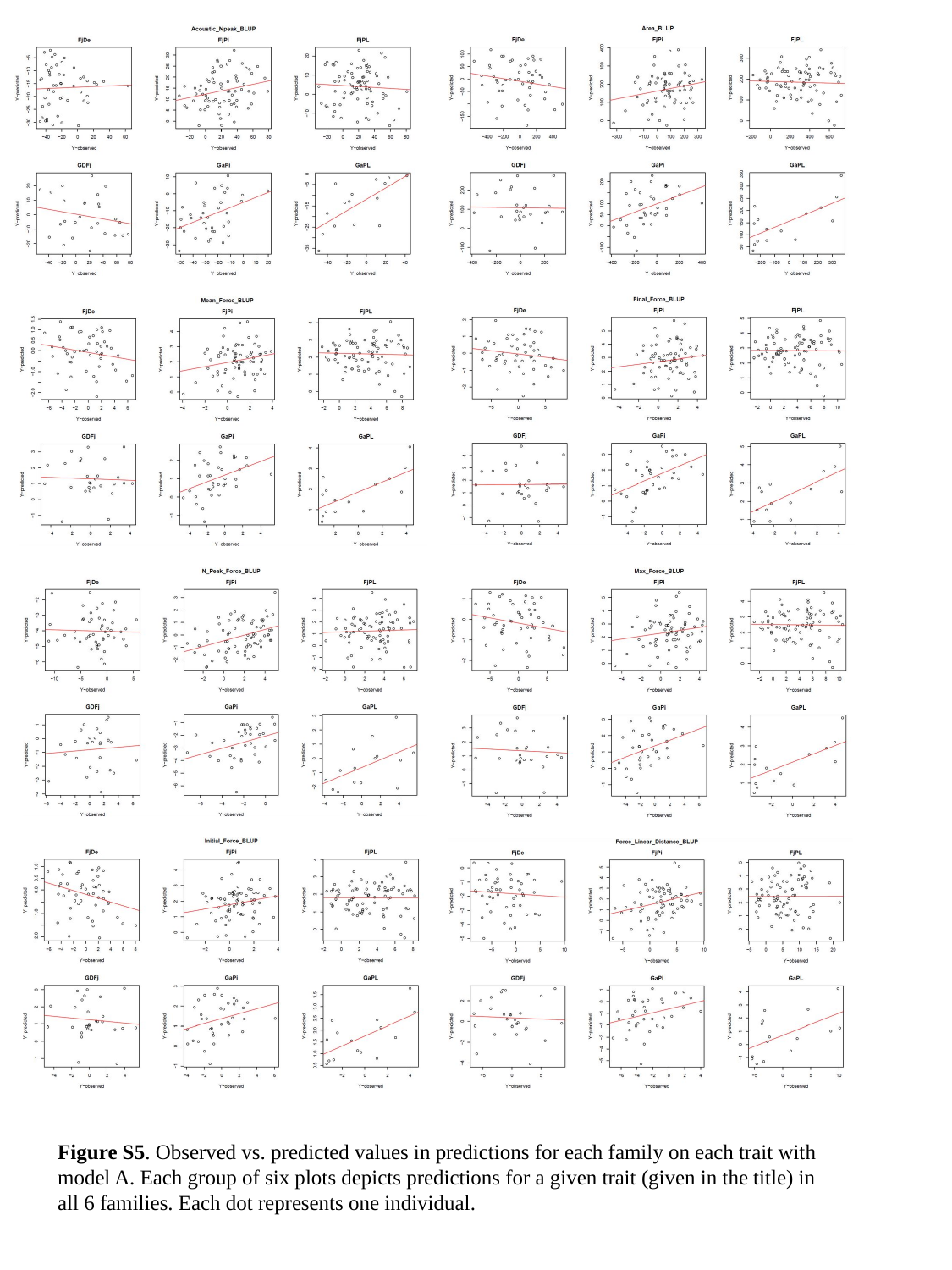

Figure S5. Observed vs. predicted values in predictions for each family on each trait with model A. Each group of six plots depicts predictions for a given trait (given in the title) in all 6 families. Each dot represents one individual.

## Slide 6
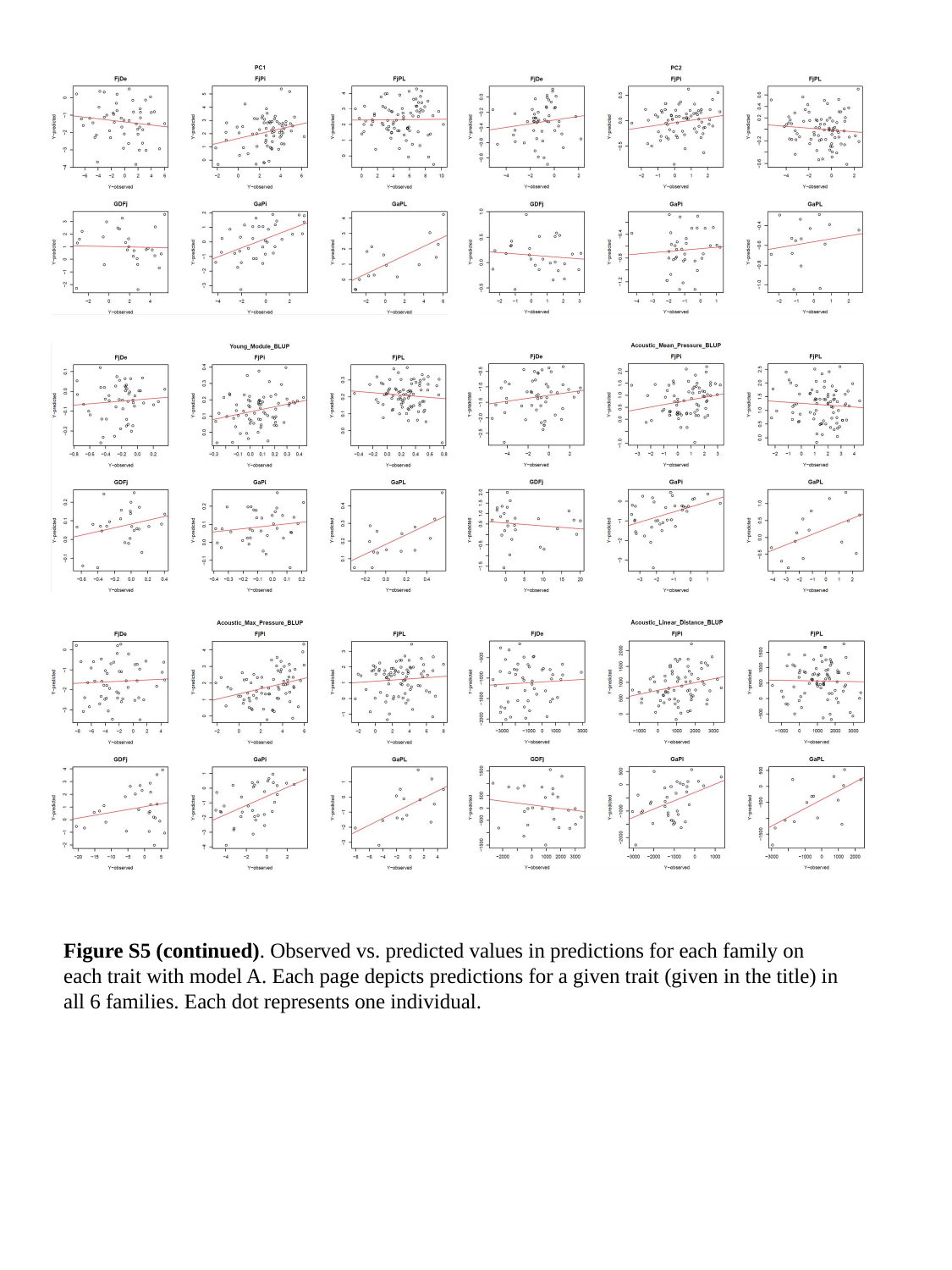

Figure S5 (continued). Observed vs. predicted values in predictions for each family on each trait with model A. Each page depicts predictions for a given trait (given in the title) in all 6 families. Each dot represents one individual.

## Slide 7
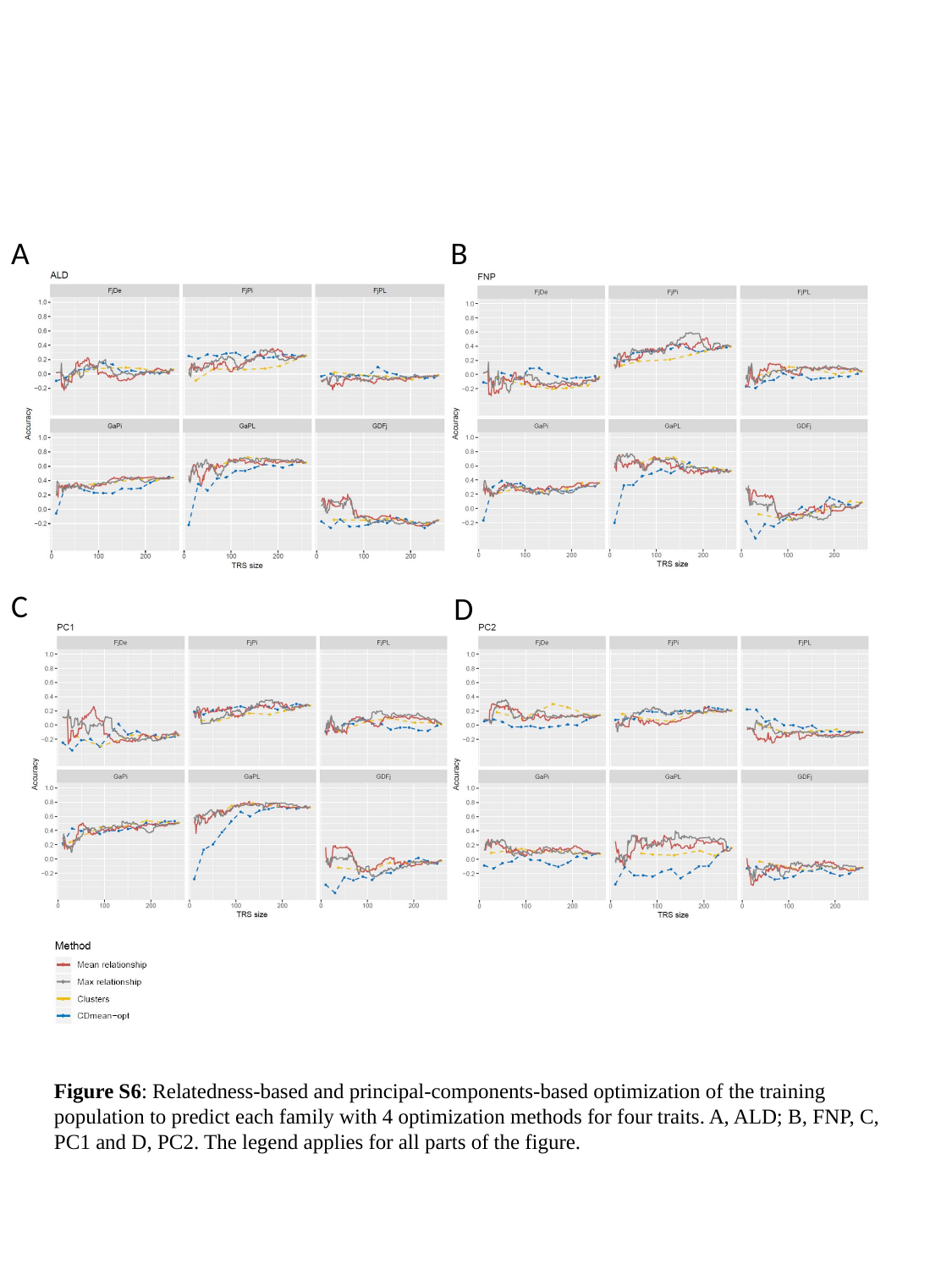

A
B
C
D
Figure S6: Relatedness-based and principal-components-based optimization of the training population to predict each family with 4 optimization methods for four traits. A, ALD; B, FNP, C, PC1 and D, PC2. The legend applies for all parts of the figure.
